# The Preliminary Evaluation of Epigenetic Modifications Regulating the Expression of *IL10* in Insulin-Resistant Adipocytes

**DOI:** 10.3390/genes13020294

**Published:** 2022-02-02

**Authors:** Aneta Cierzniak, Krzysztof Kaliszewski, Małgorzata Małodobra-Mazur

**Affiliations:** 1 Department of Forensic Medicine, Department of Molecular Techniques, Wrocław Medical University, Skłodowskiej-Curie 52, 50-369 Wroclaw, Poland; aneta.cierzniak@umw.edu.pl; 2 Department of General, Minimally Invasive and Endocrine Surgery, Wrocław Medical University, Borowska 213, 50-556 Wroclaw, Poland; krzysztof.kaliszewski@umw.edu.pl

**Keywords:** *IL10* expression, insulin resistance, epigenetic modifications

## Abstract

A higher level of *IL10* expression in obesity and insulin resistance was observed in both human and mouse WAT. In our research, we analyzed the influence of insulin resistance on epigenetic modification within the promoter region *IL10* gene and the potential influence of these modifications on its expression. Studies were performed using two cell models for the analysis: human, preadipocytes derived from adipose (visceral and subcutaneous) tissues and murine 3T3-L1 fibroblasts. We demonstrated a significant increase in the *IL10* expression level, *IL10* promoter region methylation, and histone 3 epigenetic modifications: H3K4me and H3K9/14ac, in insulin resistance cells (IR) from SAT cell culture. In IR cells from VAT cell culture, we observed decreased *IL10* expression with a simultaneous increase of *IL10* promoter region methylation. In IR cells from 3T3L1 cell culture, we observed the increased expression of *IL10* as well as the decreased levels of methylation in the *IL10* promoter region and histone methylation (H3K4me) and acetylation (H3K9/14ac). The presented analyses suggest a potential impact of epigenetic modifications on gene expression and a potential mutual influence of epigenetic modifications on each other or the activation of specific epigenetic regulation at a different stage of the development of insulin resistance in cells.

## 1. Introduction

The excessive accumulation of adipose tissue is associated with environmental, metabolic, psychological, endocrine and genetic factors. The process of excessive adipogenesis causes changes in metabolism and endocrine function, which can lead to an increased release of hormones, fatty acids and proinflammatory factors that contribute to metabolic disorders. Obesity is considered to be the strongest risk factor predisposing patients to insulin resistance [1,2]. One of the pathomechanisms that could explain the relationship between obesity and insulin resistance is chronic inflammation. Obesity is characterized by altered levels of circulating cytokines, adipose tissue macrophage accumulation, and inflammation state [3,4]. Studies conducted on both murine and human tissues indicate an increased accumulation of proinflammatory factors and cells in white adipose tissue (WAT) [5,6,7].

Interleukin-10 (IL-10) is considered an anti-inflammatory cytokine that inhibits the production of such proinflammatory factors as TNF-α, IL-2, IL-3, IL-6. IL-10 is produced mainly by macrophages, dendritic cells, B lymphocytes, and T lymphocytes [8,9]. Moreover, it is also secreted by the mature adipocyte fraction of human WAT [10,11].

A higher level of *IL10* expression was observed in obesity and insulin resistance subjects [10,11,12]. What is more the current scientific reports suggest that IL-10 could have a protective effect in the obesity-related development of insulin resistance in some tissue, including skeletal muscle [13,14,15]. This protective effect of IL-10 was associated with a reduction in local cytokine expression in skeletal muscle and macrophage levels in mice [13]. The exact mechanism of the influence of IL-10 on the improvement of cell insulin sensitivity is at the moment unclear, however, it is suggested that it is directly related to IL-10 anti-inflammatory activity manifested by promoting an anti-inflammatory phenotype of macrophage [16]. On the other hand, Lumeng et al. suggested that the therapeutic effect of IL-10 is associated with the protection of adipocytes against the physiological effects of TNF-α causing insulin desensitization observed in obesity [17]. Given the potential therapeutic value of this cytokine, it is important to research the mechanism of the expression of this gene in insulin resistance and obesity.

Obesity and insulin resistance are characterized by severe long-term metabolic changes, based on alteration in gene expression, which occur throughout life due to external factors (high-fat diet, sedentary lifestyle, stress). Therefore, it is highly appropriate to look for the ground for these epigenetic regulation changes, both at the DNA and histone levels [18]. DNA methylation is a significant epigenetic modification; it is mostly associated with transcriptional repression. The histone modification effect on gene expression is directly related to the location of methylation and acetylation within the histone. These epigenetic regulation modifications can be both silencing and transcription stimulating.

In our research, we analyzed the influence of insulin resistance on epigenetic modification (both at the DNA and histone levels) within the promoter region of the gene encoding IL-10 (*IL10*) as well as the potential influence of these modifications on *IL10* expression. Studies were performed using two cell models for the analysis: human preadipocyte, obtained from the previously collected adipose tissues (visceral and subcutaneous), and murine 3T3-L1 cell line. The use of adipocytes in the study is essential in the analysis of potential relationships between obesity and insulin resistance. Adipose cells are the most representative in this type of analysis because WAT has the ability to self-produce IL-10.

## 2. Materials and Methods

The research protocols and all procedures were approved by the Ethical Review Board of Wroclaw Medical University (approval No. KB-124/2017).

### 2.1. Human and Mouse Cell Culture

Human preadipocytes were extracted from both subcutaneous and visceral adipose tissues obtained from three healthy subjects with BMI 20–25 kg/m^2^ and proper insulin sensitivity, aged between 40–60 years old. All enrolled patients were men. Adipose tissues were collected during abdominal surgery in the Department of General, Minimally Invasive and Endocrine Surgery of Wroclaw Medical University, following a written agreement. Informed consent was obtained from all subjects involved in the study. Each patient completed a questionnaire regarding metabolic diseases (type 2 diabetes, hypertension, sclerosis) and medications. The mean age of patients was 45.

Adipose tissue biopsies were transported to the Molecular Technique Unit in PBS enriched with PI (protein inhibitors, BioShop, Burlington, ON, Canada). Each tissue (SAT and VAT) was treated according to the following procedure. In the laboratory, tissue was cleaned of blood vessels and cut with scissors. The fragmented tissue was completely digested with collagenase (Sigma-Aldrich, St. Louis, MO, USA) (1 mg/mL) with the addition of BSA (Bovine Serum Albumin, Sigma-Aldrich, St. Louis, MO, USA) (10 mg/mL). After centrifugation (2000 rpm, 5 min), the supernatant was discarded and the cell pellet was washed twice with ice-cold PBS and then once with DMEM/F12 (50:50, Dulbecco’s Modified Eagle Medium/Nutrient Mixture F-12, Corning, New York, NY, USA) supplemented with antibiotics (50 µg/mL of streptomycin and 50 U/mL of penicillin, Corning, New York, NY, USA). The cells were suspended in DMEM/F12 enriched with 10% FCS (Fetal Calf Serum, Sigma-Aldrich, St. Louis, MO, USA) and antibiotics after the final centrifugation,

Commercial mouse fibroblasts of the 3T3-L1 (ATCC, CRL-3242™, University Blvd. Manassas, VA, USA) cell line were used for mouse cell culture.

Mouse cell line and VAT/SAT primary cultures have been tested and found free of Mycoplasma.

### 2.2. Human and Mouse Cell Line Culture, Differentiation and Insulin Resistance Induction

Both types of cells were cultured in a humidified incubator at 37 °C and 5% CO_2_ (Heracell 240i incubator, Thermo Scientific, Waltham, MA, USA).

The human cell line was cultured with medium contained DMEM/F12 (Corning, New York, NY, USA), 10% FCS (Sigma-Aldrich, St. Louis, MO, USA) and antibiotics (penicillin, 50 U/mL; streptomycin, 50 µg/mL, Corning, New York, NY, USA) until it became 100% confluent.

The 3T3-L1cell line was cultured with medium contained DMEM (Dulbecco’s Modified Eagle Medium, Corning, New York, NY, USA), 10% FCS (Sigma-Aldrich, St. Louis, MO, USA), and antibiotics (penicillin, 50 U/mL; streptomycin, 50 µg/mL, Corning, New York, NY, USA) until it became 100% confluent.

The human and 3T3-L1 cell cultures were differentiated after the achievement of 100% confluence. The human cells were cultured in the differentiation medium containing 10% FCS (Sigma-Aldrich, St. Louis, MO, USA), DMEM/F12, antibiotics (penicillin, 50 U/mL; streptomycin, 50 µg/mL, Corning, New York, NY, USA), dexamethasone (390 ng/mL, Sigma-Aldrich, St. Louis, MO, USA), 3-isobutyl-1-methylxanthine (115 µg/mL, Sigma-Aldrich, St. Louis, MO, USA), human transferrin (10 µg/mL, Sigma-Aldrich, St. Louis, MO, USA), insulin (10 µg/mL, Sigma-Aldrich, St. Louis, MO, USA) and pioglitazone hydrochloride (0.1 µg/mL, Sigma-Aldrich, St. Louis, MO, USA) for three days. Next, the medium was changed to DMEM/F12 with antibiotics, 10% FCS, human transferrin (10 µg/mL), and insulin (10 µg/mL). After three more days, the medium was changed to DMEM/F12 with antibiotics, 10% FCS, and further cultured for additional two days to achieve the fully mature phenotype. All three sets of human adipocytes (VAT and SAT derived cells) were run in two independent experiments.

The 3T3-L1 cells were cultured in the differentiation medium containing DMEM, 10% FBS (Corning, New York, NY, USA), antibiotics (penicillin, 50 U/mL; streptomycin, 50 µg/mL, Corning, New York, NY, USA), 3-isobutyl-1-methylxanthine (115 µg/mL, Sigma-Aldrich, St. Louis, MO, USA), dexamethasone (390 ng/mL, Sigma-Aldrich, St. Louis, MO, USA) and insulin (10 µg/mL, Sigma-Aldrich, St. Louis, MO, USA) for three days. Next, the medium was changed to DMEM with antibiotics, 10% FBS, and insulin (10 µg/mL). After three more days, the medium was changed to DMEM with antibiotics, 10% FBS, and further cultured for additional two days to achieve the fully mature phenotype. The cell culture was done in three independent experiments.

The process of preadipocyte differentiation into mature adipocytes has previously been optimized and published [19,20]. The progress of the adipogenesis process was controlled by lipids accumulation measurement and morphological changes. The cells were recorded using Olympus IX81 before differentiation and at the end of adipogenesis (Figure S1). Lipids accumulation was measured using Oil Red O stain (Sigma-Aldrich, St. Louis, MO, USA) (Figure S2). The cells were incubated with 4% paraformaldehyde (Sigma-Aldrich, St. Louis, MO, USA) for 10 min, with 60% isopropanol (Sigma-Aldrich, St. Louis, MO, USA) for 5 min, and with the previously prepared Oil Red O solution for 30 min. All incubations were performed at room temperature. The accumulated Oil Red O in the cells was extracted with 100% isopropanol and the absorbance was measured at 492 nm.

### 2.3. Insulin Resistance Induction

When the adipose cells were completely differentiated, insulin resistance was induced by 0.5 mM palmitic acid (16:0) (Sigma-Aldrich, St. Louis, MO, USA). Insulin resistance was stimulated for 48 h and 72 h using palmitic acid. After each time point, the glucose uptake test was performed to assess the insulin resistance state of the mature adipocytes. Glucose uptake was analyzed using Glo-Glucose Uptake (Promega, Madison, WI, USA) according to the manufacturer’s protocol. Luminescence was read using a Victor3 1420 Multilabel Counter.

### 2.4. DNA and RNA Isolation

DNA from the cells from the cell cultures was isolated using the phenol:chloroform method (Sigma-Aldrich, St. Louis, MO, USA). Total RNA from the cells from the cell cultures was isolated through the trizol method (Sigma-Aldrich, St. Louis, MO, USA).

### 2.5. Reverse Transcription Reaction and Gene Expression Level

Reverse transcription was performed using isolated total RNA (200 ng) and a High Capacity cDNA Reverse Transcription Kit (Applied Biosystems, Waltham, MA, USA). Gene expression was done using real-time PCR based on an SYBR Green assay (Applied Biosystems, Waltham, MA, USA). Primers to *IL10* and β-actin (*ACTB*) were manually designed to flank two mRNA exons (Table 1). The specificity of primers was checked using Primer-BLAST; secondary structures were analyzed using OligoAnalyzer. Prior to real-time PCR, the efficiency of the primers was analyzed using the standard curve method. Specificity was checked based on the denaturation curve. Only primers characterized by efficiency values higher than *R^2^* ≥ 0.92 were used for gene expression studies. A relative gene expression level, normalized to the housekeeping gene β-actin, was calculated using the delta-delta Ct (ΔΔCt) model.

### 2.6. Site-Specific DNA Methylation of Cells from Cell Cultures

The DNA methylation of the cells from the cell cultures was analyzed using MagMeDIP qPCR Kit (Diagenode, Denville, NJ, USA) according to the manufacturer’s protocol. After precipitation and DNA extraction, DNA concentration was measured, using Pico488 dsDNA quantification reagent (Lumiprobe, Cockeysville, MD, USA). The standard curve was prepared from human DNA quantified by Quantifiler™ Duo DNA Quantification Kit (Thermo Fisher, Waltham, MA, USA). The global DNA methylation was quantified as the percentage of DNA immunoprecipitated using C-me antibodies (Diagenode, Denville, NJ, USA) to the input amount of DNA, based on the concentration results. The site-specific DNA methylation was analyzed in Real-Time PCR, using Fast SYBR Green Master Mix (Thermo Fisher, Waltham, MA, USA). The primer sequences are presented in Table 1. The promoter region of the *IL10* gene was the target of primers. The calculation of % of the input was done according to the manufacturer’s protocol. The following formula was used:

% of recovery = 2^(CtInput − 3.32 − CtCme)^*100,

where, CtInput—Ct value of 10% Input; CtCme—Ct value of precipitated DNA using Cme antibody.

### 2.7. Global and Site-Specific Histone Modifications

The cultured cells were treated with 1% formaldehyde (FA, Sigma-Aldrich, St. Louis, MO, USA) with the addition of PI (BioShop, Burlington, ON, Canada) and PMSF (phenylmethylsulfonyl fluoride, Sigma-Aldrich, St. Louis, MO, USA) (1 mM) for ten minutes at 37 °C. After ten minutes, the reaction was stopped by Glycine. Next, the cells were washed three times with PBS with PI and PMSF. The cells were scraped and suspended in 300 µL of SDS Lysis Buffer supplemented with PI and PMSF and sonicated ((30 s ON, 45 s OFF) × 15). The next steps of the procedure were carried out according to the manufacturer’s protocol of the Chromatin Immunoprecipitation (ChIP) Assay Kit (Millipore, Burlington, MA, USA).

The H3K4me3, H3K9/14ac, and IgG rabbit antibodies were purchased from Diagenode (Denville, NJ, USA)and added at the precipitation stage according to the manufacturer’s protocol.

After decross-link by adding 20 µL of 5 M NaCl, DNA was extracted as described in Section 2.3.

The ChIP-Real-Time PCR was done as described in Section 2.5. Primers for real-time PCR were designed manually (Table 1). The promoter region of the *IL10* gene was the target of primers. The results were calculated as the percentage of input (% of input) according to the following formula:

% of Input = 100 × 2^(CtAI-CtIP)^

where CtAI—Ct value of adjusted Input (calculated as Ct value—6.644), CtIP—Ct value of IP samples.

The results of the sonication process optimization are presented in Figure S3.

### 2.8. Statistical Analysis

Statistical analysis was performed using STATISTICA 13.1 (TIBCO Software Inc, Palo Alto, CA, USA) and Microsoft Office Excel 2007 (Microsoft, Redmond, WA, USA). The differences between the studied groups were performed using Student’s T-Test. The correlation between numerical values was made using the Pearson correlation coefficient. Statistical significance was set at *p* < 0.05.

Statistical analysis was performed based on the results obtained from three replicate experiments for the mouse culture and the results obtained from three different human cell cultures.

## 3. Results

The analyses were performed at two time points, 48 and 72 h after the induction of insulin resistance by palmitic acid (16:0). In the experimental cells after culturing with 0.5 mM of palmitic acid (16:0), the insulin resistance state was developed, confirmed by the glucose uptake test. After 10 min insulin stimulation (1 µM), no increase in insulin-stimulated glucose uptake by those cells was observed. Control cells remained insulin-sensitive; insulin caused at least a twofold increase in glucose uptake compared to basal glucose utilization. The results have been shown by us in the recent publication [21].

### 3.1. IL10 Expression Level

**Figure 1:**
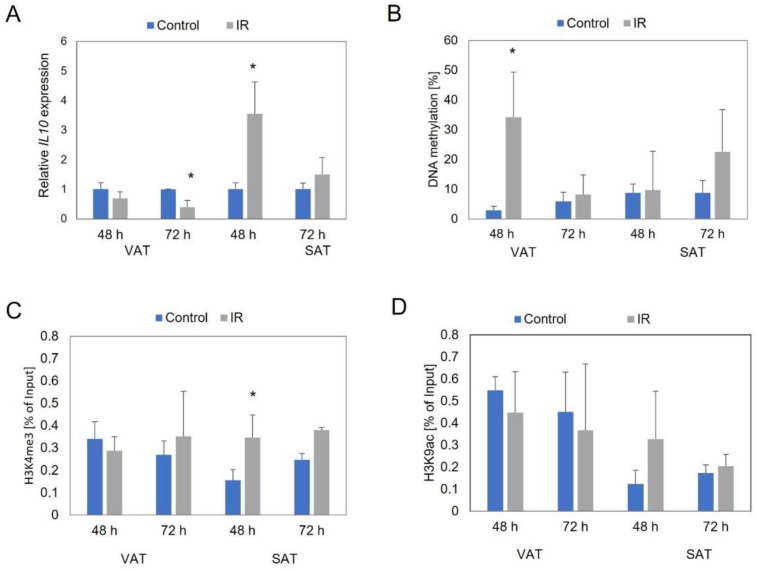
The *IL10* expression (**A**), DNA methylation (**B**), histone methylation (**C**), and histone acetylation level (**D**) in visceral (VAT) and subcutaneous (SAT) derived control adipocytes and insulin resistant adipocytes (IR) after 48 and 72 h after insulin resistance induction. * *p* < 0.05.

In the human VAT-derived adipocytes, we observed a 1.5-fold decrease in *IL10* expression in insulin-resistant cells (IR), compared to control cells (*p* = 0.169) after 48 h after IR induction, and a 2.5-fold decrease after 72 h (*p* = 0.002) (Figure 1A).

In the human SAT-derived adipocytes, we observed a 3.5-fold increase in *IL10* expression in IR cells, compared to control cells after 48 h after insulin resistance induction (*p* = 0.011), and a 1.5-fold increase after 72 h (*p* = 0.137) (Figure 1A).

**Figure 2:**
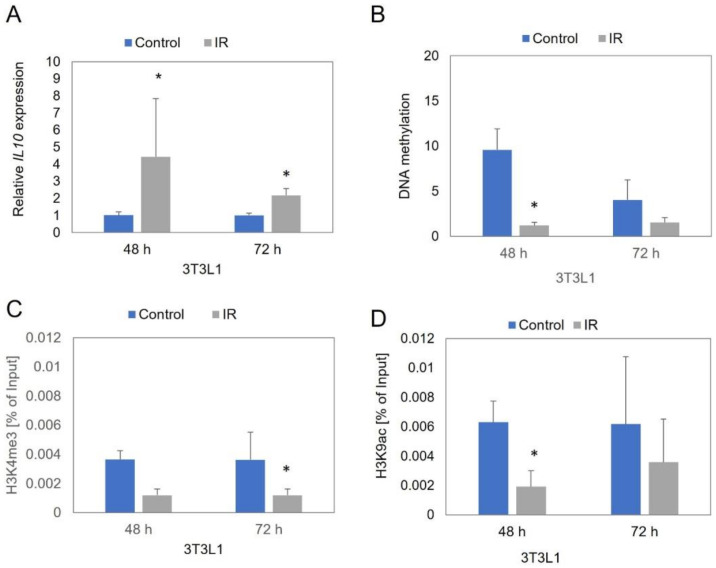
The *IL10* expression (**A**), DNA methylation (**B**), histone methylation (**C**), and histone acetylation level (**D**) in control 3T3L1 adipocytes and in insulin resistant (IR) adipocytes after 48 and 72 h after insulin resistance induction. * *p* < 0.05.

In 3T3-L1 IR cells, similar to the SAT-derived IR adipocytes, we observed approximately a 4.5-fold increase of the *IL10* expression level in IR cells, compared to control cells after 48 h after induction insulin resistance (*p* = 0.014), and more than a twofold increase after 72 h (*p* = 0.001) (Figure 2A).

### 3.2. IL10 Promoter Region Methylation Level

The site-specific DNA methylation analyses showed in both the VAT and SAT human adipocytes an increase in the *IL10* promoter region methylation level (assessment based on % of the input). In the VAT samples, the *IL10* promoter was almost twelve times more highly methylated in IR cells, compared to control cells (*p* = 0.006) after 48 h of IR induction and a slight increase in *IL10* promoter methylation after 72 h (Figure 1B). In the SAT-derived adipocytes, we noticed a slight increase in *IL10* promoter methylation after 48 h in IR cells, compared to control cells, and a 2.5-fold increase after 72 h (*p* = 0110) (Figure 1B).

However, the analysis of 3T3-L1 cells showed a decrease of the *IL10* promoter region methylation level in IR cells compared to control cells. After 48 h, we observed an almost eightfold decrease of methylation in IR cells (*p* = 0.000), and more than a 2.5-fold decrease after 72 h (*p* = 0.073) (Figure 2B).

### 3.3. Site-Specific Histone Modifications near IL10 Gene

In the human VAT adipocytes, we did not observe statistically significant epigenetic changes within the histones at both time points. (Figure 1C,D).

We observed significant changes in the level of histone modification in human adipocytes collected from SAT. The increased methylation (H3K4me3; more than twofold increase; *p* = 0.025) and acetylation of histone 3 (H3K9/14ac; more than 2.5-fold increase; *p* = 0.279) were shown in IR cells, compared to control cells after 48 h. At the second time point (72 h), we also observed increases of these modifications in IR cells but these increases were smaller (H3K4me3: *p* = 0.078; H3K9/14ac: *p* = 0.279) (Figure 1C,D).

In 3T3-L1 IR cells, we observed a decreased histone modification level, compared to control cells both after 48 and 72 h of IR induction in experimental cells. We observed more than a threefold decrease of the methylation and acetylation level of histone 3 after 48 h (H3K4me3: *p* = 0.094; H3K9/14ac: *p* = 0.003) and a threefold decrease of methylation and almost a twofold decrease of acetylation in IR cells (H3K4me3: *p* = 0.047; H3K9/14ac: *p* = 0.379) (Figure 2C,D).

### 3.4. Correlation Analysis

At the first time point (48 h), in VAT derived adipocytes we observed positive correlations between *IL10* expression and H3K9/14 enrichment (R = 0.6812; *p* = 0.136). At the second time point (72 h), we observed negative correlations between *IL10* expression and the histone 3 methylation level (R = −0.6196; *p* = 0.138). We also observed a correlation between measured epigenetic modifications. A positive correlation between H3K4me3 and H3K9/14 enrichment was observed at both time points (48 h: R = 0.5735; *p* = 0.137; 72 h: R = 0.4839; *p* = 0.271). After 72 h we observed also negative correlation between *IL10* promoter region methylation and histone 3 epigenetic modifications (H3K4me3: R = −0.6372; *p* = 0.124; H3K9ac: R = −0.5252; *p* = 0.181).

After 48 h in SAT-derived adipocytes we showed positive correlations between *IL10* expression and both histone modifications (H3K4me3: R = 0.6693; *p* = 0.146; H3K9ac: R = 0.8778; *p* = 0.021). At the second time point, we showed positive correlations between *IL10* expression and histone 3 methylation level (R = 0.5371; *p* = 0.272). At the first time point we observed negative correlations between *IL10* promoter methylation level and histone methylation (H3K4me3: R = −0.9015; *p* = 0.037). After 72 h we observed positive correlation between *IL10* promoter region methylation and histone 3 methylation level (R = 0.6513; *p* = 0.161), and negative correlation between *IL10* promoter region methylation and histone 3 acetylation (R = −0.6484; *p* = 0.115).

In the case of 3T3-L1 adipocytes, at the first time point, we observed a negative correlation between *IL10* expression and the histone methylation level (R = −0.6842; *p* = 0.061), at the second time point, we observed a positive correlation between the expression level of *IL10* and histone 3 acetylation level (R = 0.8949; *p* = 0.016). After 48 h we observed positive correlations between methylation of the *IL10* promoter region and histone 3 methylation, and also histone 3 acetylation (H3K4me3: R = 0.4210; *p* = 0.299; H3K9ac: R = 0.8333; *p* = 0.010). After 72 h we showed positive correlation between histone 3 acetylation and histone 3 methylation (R = 0.4767; *p* = 0.232). The summarizing results of correlation analysis are presented in Table 2.

## 4. Discussion

Insulin resistance and obesity belong to serious epidemiological problems in the world. However, the only clinically used drug that increases the sensitivity of cells to insulin is metformin; however, long-term treatment decreases its efficiency. Given that these metabolic disorders constitute a serious problem in modern society, affecting children and adults, it is important to find effective treatments. Scientific reports indicating the effect of improving insulin sensitivity by IL-10 provide grounds for further research on this cytokine [13,14,15]. In the present study, we analyzed the influence of insulin resistance on epigenetic modification in the promoter region of the *IL10* gene as well as the potential influence of these modifications on *IL10* expression in adipocytes.

Studies were performed using two independent cell models for the analysis: human cells, based on mesenchymal stem cells collected from stromal fraction of white adipose tissue obtained from previously collected adipose tissues (visceral and subcutaneous), and murine cell line, based on the commercial 3T3-L1 cell line. The cells of both cultures were differentiated in the adipogenesis process into mature adipocytes. The use of adipocytes in the study is essential in the analysis of potential relationships between obesity and insulin resistance. Adipose cells are the most representative in this type of analysis, given the fact that WAT has the ability to self-produce IL-10. Cellular insulin resistance was induced with the use of palmitic acid. This is one of the most popular methods for diet-induced insulin resistance cellular models [22].

The increase in the *IL10* expression level in cells with induced insulin resistance, which we observed in the subcutaneous human cell culture in first time point (after 48 h), confirms a previous scientific report [11]. The increase in *IL10* expression is probably the result of the inflammation processes occurring during the development of insulin resistance in adipocytes. It could be a cell defense mechanism against intensifying inflammation. The developing inflammation accompanying the development of insulin resistance in cells may be a factor stimulating the increase in the expression of anti-inflammatory cytokines such as IL-10. We observed an increase of the *IL10* expression level in IR cells from the SAT cell culture at both time points of the experiment. However, the strength of these increases is different. After 48 h, we observed a 3.5-fold increase but after 72 h, it was only 1.5-fold, which probably indicates the process of expression inhibition. We only obtained statistical significance at the first time point.

Changes in the expression level at time points may be directly related to the changes in methylation levels. The increase in methylation after 48 h was insignificant. However, after 72 h, it is already two and a half times, which could explain the decreasing level of expression in these cells. This observation could confirm the transcription silencing effect of DNA methylation [23]. However, the above-mentioned hypothesis needs to be confirmed experimentally, especially since we didn’t obtain the statistical significance of these results from both time points.

The initial increase in *IL10* expression at the first time point should also be referred to the histone 3 methylation analysis. After 48 h, we observed more than a double increase of H3K4me3, compared to the control, which also positively correlated with the increased expression of *IL10*. Acetylation of histone 3 in IR cells also be increased but the results didn’t show a statistical significance, however we observed a strong positive correlation between *IL10* expression and H3K9/14ac with statistical significance. This observation could confirm the transcription activating effect of H3K4me3 and H3K9/14ac [24]. After 72 h, we observed a reduced increase of histone modifications, compared to the first time point, which could explain the simultaneous decrease in expression, however, this experiment should be repeated given the lack of statistical significance in the results obtained.

With a reduced increase of histone modifications, we also observed an increase in *IL10* promoter region methylation. A similar linkage between H3K4me3 and DNA methylation has been observed by others. It has been shown that these modifications tend to be mutually exclusive. The role in this mechanism was assigned to the DNMT3L protein. These data indicate that DNMT3L recognizes histone H3 tails that are unmethylated at lysine 4 and induces de novo DNA methylation by the recruitment or activation of DNMT3A2 so—in this way—nonmethylated H3K4 could support DNA methylation [25]. Our observations suggest that these two epigenetic mechanisms may have a compensatory effect on each other, as shown by the strong negative correlation we observed between promoter methylation and H3K4me3 after 48 h, and between promoter methylation and H3K9/14ac after 72 h.

In the case of the visceral human cell culture, at the first time point, we observed a 1.5-fold decrease in expression of *IL10* in IR cells, and after 72 h, it was already a 2.5-fold decrease. At the same time, we observed an increase in methylation of the promoter region of *IL10* in these cells. After 48 h, the increase in IR DNA methylation was as much as twelvefold. After 72 h it was only 1.5-fold and didn’t show statistical significance. The demonstrated significant increase in methylation of the promoter region of *IL10* after 48 h could directly impact a significant reduction in gene expression, which was observed after 72 h. In turn, the change in the increase in DNA methylation between these two time points may result from the changes in the level of H3K4me3 observed by us. We showed a negative correlation between the methylation of the promoter region of *IL10* and the level of H3K4me3. We observed a significant increase in DNA methylation (twelve-fold) with a simultaneous decrease in H3K3me3 after 48 h and a decreased power of the increase in DNA methylation (only 1.5-fold) with a simultaneous increase in histone methylation after 72 h. It could confirm an inverse relationship between these epigenetic modifications. However, unlike the SAT cell culture, DNA methylation decreased with increasing methylation within histone 3. In a recent study, it was observed that DNMT3L can bind to nonmethylated H3K4 but cannot bind to H3K4me3. It was suggested that H3K4 methylation can play a role in blocking de novo DNA methylation at some genomic loci [26].

Different expression profiles of *IL10* in VAT and SAT cell cultures may directly result from the metabolic differences between these two adipose tissue types. As has been shown before by us, VAT is more susceptible to developing chronic inflammation and shows an increased expression of proinflammatory factors (IL-6 and TNFα), compared to SAT [19]. The increased expression of proinflammatory factors may be the reason for the downregulation of *IL10* expression.

Observations in the mouse cell model, as well as in the SAT human cell culture, showed a strong 4.5-fold increase in *IL10* expression in IR cells 48 h after the induction of insulin resistance and twofold after 72 h. At the same time, we observed a decrease in the degree of methylation of the *IL10* promoter region in IR cells, which would explain the changes in expression observed. Methylation levels dropped eightfold at the first time point and by two and a half times at the second time point. The increase in methylation in IR cells between 48 and 72 h would explain the reduction in the level of *IL10* expression.

The analysis of histone epigenetic modifications showed decreased levels of both H3K4me3 and H3K9/14ac in IR cells at both time points. We observed a strong negative correlation between *IL10* expression and histone methylation at the first time point. On the other hand, after 72 h, we showed a strong positive correlation between *IL10* expression and histone acetylation. This observation could suggest a different effect on the transcription activity of both histone modifications in the mouse model. According to the authors’ knowledge, this is the first scientific report related to the epigenetic regulation of *IL10* expression in 3T3L1 cells.

The presented study has a few limitations. First of all, the amount of human adipose tissue samples used to isolate MSC is relatively low, and in the study were used only male-derived human cells. Another limitation that should be discussed is the method of inducing insulin resistance in cells. Bearing in mind the fact that the protocol for inducing insulin resistance with palmitic acid is usually used in cell culture studies, it should be remembered that in vivo this process is much more complicated and generated by more than one factor. Furthermore, epigenetic regulation is a complex process, which gives difficulties in the interpretation of single regulation. Unfortunately, the cell culture model itself has limitations due to the lack of influence of other organs, such as the liver or pancreas, on the development of metabolic disorders. Therefore, it would be important to check the mechanisms described in the paper in vivo with the use of adipose tissue samples.

The analyses presented in this study suggest a potential impact of epigenetic modifications on gene expression and could confirm the mutual influence of epigenetic modifications on each other or the activation of specific epigenetic regulation at a different stage of the development of insulin resistance in cells. We showed that epigenetic modifications could significantly impact changes in the expression of *IL10* in adipose tissue. What is more, we presented a different expression profile of *IL10* between the SAT and VAT while confirming the metabolic dissimilarity of the two depots of adipose tissue.

## Figures and Tables

**Table 1:** The primer pairs used in a study for analysis of gene expression, site-specific DNA methylation (meDIP) and site-specific histone modifications (ChIP).

Organism	Application	Gene	Forward Sequence	Reverse Sequence	Product Length (Base Pair)	Amount of CpG Sites *
Human	Gene expression	*IL10*	GGACTTTAAGGGTTACCTGG	CTGGGTCTTGGTTCTCAGC	95	-
Human	Gene expression	*ACTB*	GAGAAGATGACCCAGATCA	TAGCACAGCCTGGATAGCAA	72	-
Human	meDIP/ChIP	*IL10*	ACTGCTCTGTTGCCTGGTC	GTCTTCACTCTGCTGAAGG	144	4
3T3L1	Gene expression	*IL10*	TAAGGGTTACTTGGGTTGCC	CGCATCCTGAGGGTCTTCA	144	-
3T3L1	Gene expression	*ACTB*	CCCAGATCATGTTTGAGACC	CTGGATGGCTACGTACATG	53	-
3T3L1	meDIP/CHIP	*IL10*	CTTGCTCTTGCACTACCAAAG	TCCTCATGCCAGTCAGTAAG	108	2

* The CpG sites are regions of DNA where a cytosine nucleotide is followed by a guanine nucleotide in the linear sequence of bases along its 5′ → 3′ direction. Cytosines in these sites can be methylated.

**Table 2:** Results of correlation analysis.

SAT		*IL10* Expression		*IL10* Promoter Region Methylation		H3K4me3		H3K9/14ac	
		48 h	72 h	48 h	72 h	48 h	72 h	48 h	72 h
*IL10* expression	48 h	-	-	-	-	r = 0.6693 *p* = 0.146		r = 0.8778 *p* = 0.021	-
	72 h	-	-	-	-	-	r = 0.5371 *p* = 0.272	-	-
*IL10* promoter region methylation	48 h	-	-	-	-	r = −0.9015 *p* = 0.037	-	-	-
	72 h	-	-	-	-	-	r = 0.6513 *p* = 0.161	-	r = −0.6484 *p* = 0.115
H3K4me3	48 h	r = 0.6693 *p* = 0.146	-	r = −0.9015 *p* = 0.037		-	-	-	-
	72 h	r = 0.5371 *p* = 0.272	-	-	r = 0.6513 *p* = 0.161	-	-	-	-
H3K9/14ac	48 h	r = 0.8778 *p* = 0.021	-	-	-	-	-	-	-
	72 h	-	-	-	r = −0.6484 *p* = 0.115	-	-	-	-
VAT		*IL10* expression		*IL10* promoter region methylation		H3K4me3		H3K9/14ac	
		48 h	72 h	48 h	72 h	48 h	72 h	48 h	72 h
*IL10* expression	48 h	-	-	-	-	-	-	r = 0.6812 *p* = 0.136	-
	72 h	-	-	-	-	-	r = −0.6196 *p* = 0.138	-	-
*IL10* promoter region methylation	48 h	-	-	-	-	-	-	-	-
	72 h	-	-	-	-	-	r = −0.6372 *p* = 0.124	-	r = −0.5252 *p* = 0.181
H3K4me3	48 h	-	-	-	-	-	-	r = 0.5735 *p* = 0.137	-
	72 h	-	r = −0.6196 *p* = 0.138	-	r = −0.6372 *p* = 0.124	-	-	-	r = 0.4839 *p* = 0.271
H3K9/14ac	48 h	r = 0.6812 *p* = 0.136	-	-	-	r = 0.5735 *p* = 0.137	-	-	-
	72 h	-	-	-	r = −0.5252 *p* = 0.181	-	r = 0.4839 *p* = 0.271	-	-
3T3L1		*IL10* expression		*IL10* promoter region methylation		H3K4me3		H3K9/14ac	
		48 h	72 h	48 h	72 h	48 h	72 h	48 h	72 h
*IL10* expression	48 h	-	-	-	-	r = −0.6842 *p* = 0.061	-	-	-
	72 h	-	-	-	-	-	-	-	r = 0.8949 *p* = 0.016
*IL10* promoter region methylation	48 h	-	-	-	-	r = 0.4210 *p* = 0.299	-	r = 0.8333 *p* = 0.010	-
	72 h	-	-	-	-	-	-	-	-
H3K4me3	48 h	r = −0.6842 *p* = 0.061	-	r = 0.4210 *p* = 0.299	-	-	-	-	-
	72 h	-	-	-	-	-	-	-	r = 0.4767 *p* = 0.232
H3K9/14ac	48 h	-	-	r = 0.8333 *p* = 0.010	-	-	-	-	-
	72 h	-	r = 0.8949 *p* = 0.016	-	-	-	r = 0.4767 *p* = 0.232	-	-

## Data Availability

Data are available on request from the corresponding author.

## Supplementary Materials

The following supporting information can be downloaded at: https://www.mdpi.com/article/10.3390/genes13020294/s1, Figure S1: title Differentiation of isolated human cells from visceral and subcutaneous adipose tissue into mature adipocytes. Visceral (A) and subcutaneous (C) preadipocytes at the start of differentiation. Visceral (B) and subcutaneous (D) matured adipocytes filled with lipid droplets upon reaching maturity; Figure S2: title The analysis of the amount of accumulated lipids measured using Oil Red O for subcutaneous (SAT) (A) and visceral (VAT) (B) cells compared to control (preadipocytes cells); Figure S3: title Results of electrophoresis in agarose gel after sonicated DNA. Lines: 1 – Size Marker (100 bp – 1000 bp; the brightest band: 500 bp); 2 – DNA isolated using MinElute PCR Purification Kit (QIAGEN) ((30s ON, 45s OFF) × 15); 3 – DNA isolated phenol:chloroform method (Sigma-Aldrich) ((30s ON, 45s OFF) × 15); 4 – DNA isolated using MinElute PCR Purification Kit (QIAGEN) ((30s ON, 45s OFF) × 20); 5 – DNA isolated phenol:chloroform method (Sigma-Aldrich) ((30s ON, 45s OFF) × 20); 6 – DNA isolated using MinElute PCR Purification Kit (QIAGEN) ((30s ON, 45s OFF) × 25); 7 – DNA isolated phenol:chloroform method (Sigma-Aldrich) ((30s ON, 45s OFF) × 25).
