# Supplementary material for: The Preliminary Evaluation of Epigenetic Modifications Regulating the Expression of IL10 in Insulin-Resistant Adipocytes
Source: Genes (Basel). 2022 Feb 2;13(2):294. doi: 10.3390/genes13020294 (PMC8872567; doi:10.3390/genes13020294)
Supplement: Supplementary file 1 [file genes-13-00294-s001.zip › genes-1529669-supplementary.pdf]

Supplemental Material

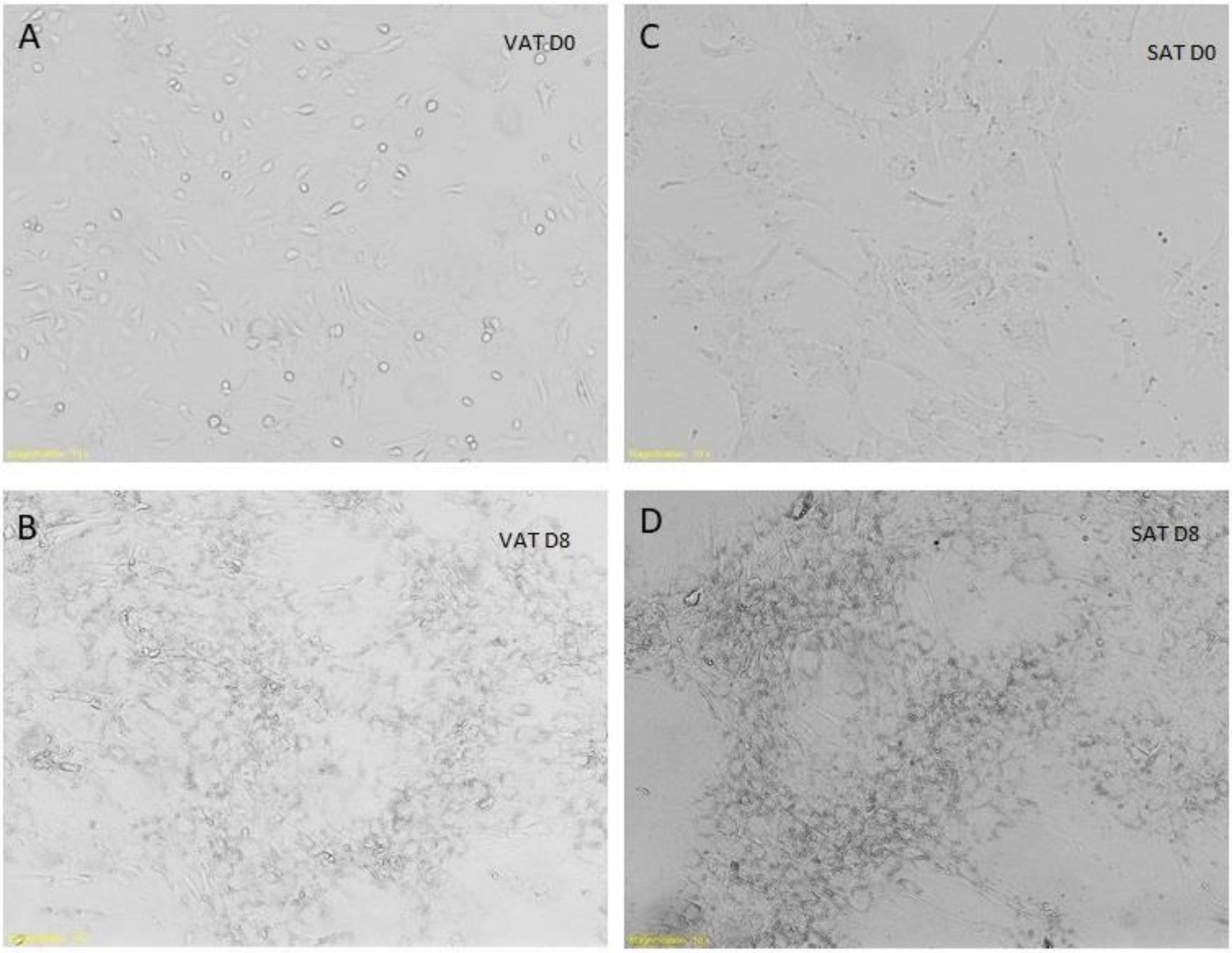

Figure S1. Differentiation of isolated human cells from visceral and subcutaneous adipose tissue into mature adipo-cytes. Visceral (A) and subcutaneous (C) preadipocytes at the start of differentiation. Visceral (B) and subcutaneous (D) matured adipocytes filled with lipid droplets upon reaching maturity.

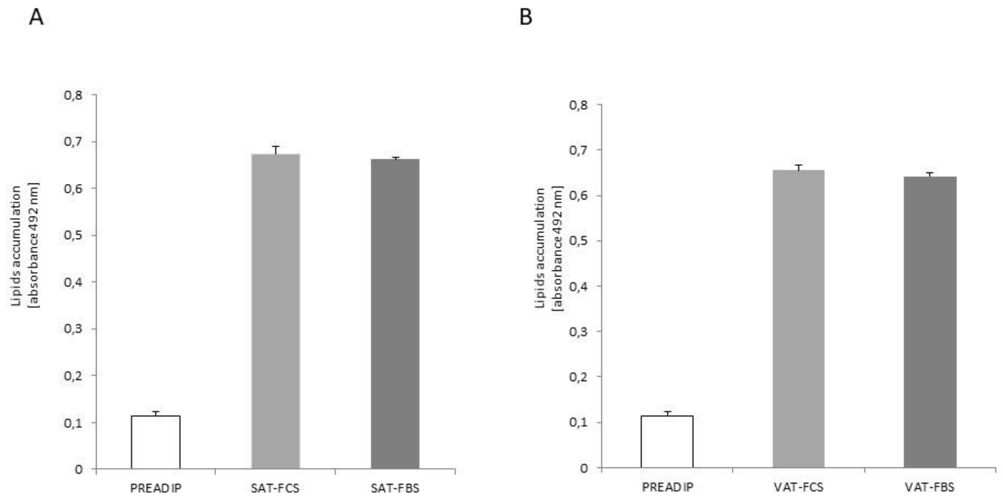

Figure S2. The analysis of the amount of accumulated lipids measured using Oil Red O for subcutaneous (SAT) (A) and visceral (VAT) (B) cells compared to control (preadipocytes cells).

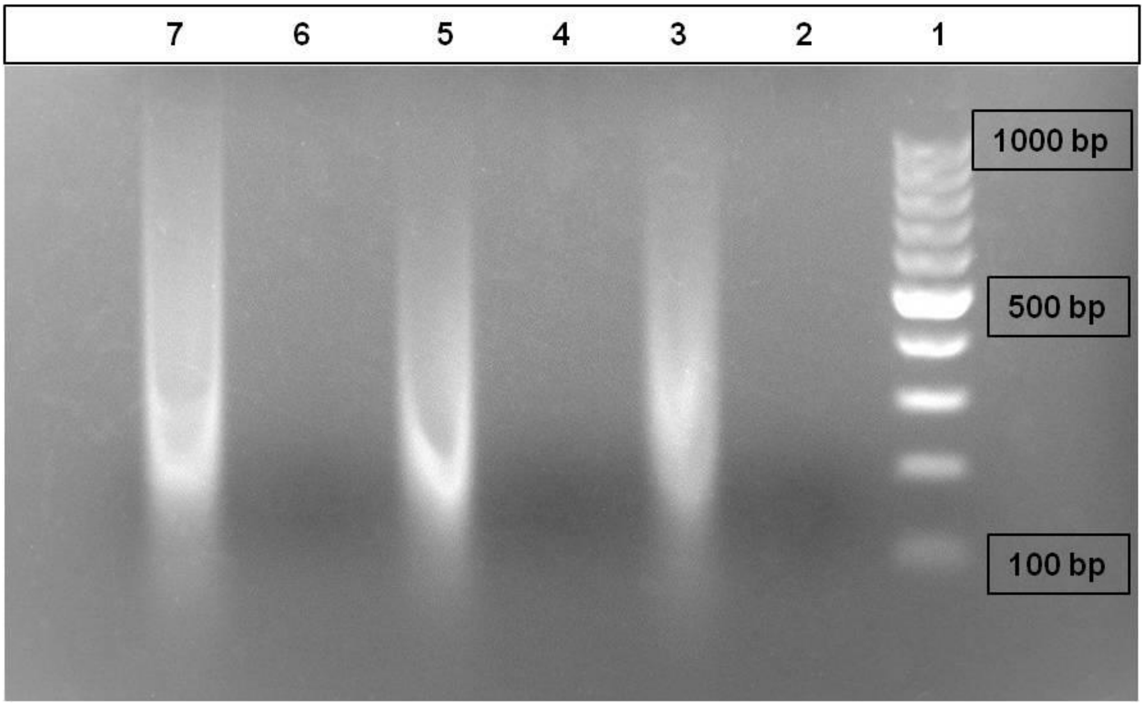

Figure S3. Results of electrophoresis in agarose gel after sonicated DNA. Lines: 1 – Size Marker (100 bp – 1000 bp; the brightest band: 500 bp); 2 – DNA isolated using MinElute PCR Purification Kit (QIAGEN) ((30s ON, 45s OFF) x 15); 3 – DNA isolated phenol:chloroform method (Sigma-Aldrich) ((30s ON, 45s OFF) x 15); 4 – DNA isolated using MinElute PCR Purification Kit (QIAGEN) ((30s ON, 45s OFF) x 20); 5 – DNA isolated phenol:chloroform method (Sigma-Aldrich) ((30s ON, 45s OFF) x 20); 6 – DNA isolated using MinElute PCR Purification Kit (QIAGEN) ((30s ON, 45s OFF) x 25); 7 – DNA isolated phenol:chloroform method (Sigma-Aldrich) ((30s ON, 45s OFF) x 25).
